# Lyme and associated tick-borne diseases: global challenges in the context of a public health threat

**DOI:** 10.3389/fcimb.2014.00074

**Published:** 2014-06-03

**Authors:** Christian Perronne

**Affiliations:** Infectious Diseases Unit, Hôpitaux Universitaires Paris-Ile de France-Ouest, Assistance Publique – Hôpitaux de Paris, University of Versailles – Saint Quentin en YvelinesGarches, France

**Keywords:** Lyme disease, *Borrelia burgdorferi*, *Borrelia miyamotoi*, novel borreliae, diagnosis, coinfections, tick borne disease, occult infection

Lyme disease, caused by *Borrelia burgdorferi* and transmitted by ticks, was initially considered a recent, rare and regional occurrence. We now have evidence that very similar bacteria infected humans in Europe during the ice age (Keller et al., 2012). Evidence-based data are scarce therefore many aspects of the disease remain controversial (Auwaerter et al., 2011; Lee and Vielmeyer, 2011; Perronne, 2012), but in 2013 the Centers for Disease Control and Prevention (CDC) revised their annual estimates from 30,000 cases to 300,000 cases in the USA alone. Having dramatically increased their numbers, the CDC are now calling Lyme disease “a tremendous public health problem in the United States” (CDC, 2011).

The lack of a gold standard for diagnosis makes producing accurate statistics difficult. Some pathogenic strains belonging to the *B. burgdorferi sensu lato* complex have a worldwide distribution, yet they are rarely considered or tested for (Varela et al., 2004; Lopes de Carvalho et al., 2009; Rudenko et al., 2009; Stanek and Reiter, 2011; Branda and Rosenberg, 2013; Clark et al., 2013; Lee et al., 2014; Margos et al., 2014). *Borrelia miyamotoi*, for instance, phylogenetically close to relapsing fever borreliae, is now recognized as a cause of Lyme-like disease and relapsing fever in Asia, Europe and North America. It usually does not cross react with *B. burgdorferi* tests (Branda and Rosenberg, 2013; Lee et al., 2014). A novel isolate of *Borrelia* has been isolated by PCR in a post-treatment serum from a patient with neurologic Lyme disease (Lee et al., 2014).

These recent historical, geographical and microbial data should prompt the medical community to realize that cases of persisting post tick-bite syndromes are probably due to multiple pathogens and that these occult infections will require a new approach if not an actual paradigm shift.

## Diagnostic pitfalls in routine practice

Classical forms of Lyme disease are usually easy to manage, but these medical conditions with pleomorphic non specific symptoms may prove confusing to physicians (Strle and Stanek, 2009). Lyme disease may mimic chronic inflammatory or degenerative diseases, including a wide range of auto-immune diseases. Although practitioners from every medical specialty are likely to have encountered cases of Lyme disease, they may have failed to recognize it, no matter how skilled they are. A major obstacle is that only 30% of the patients report a history of tick bite and only 70–80% present with a primary erythema migrans, the pathognomonic initial lesion. This lesion may go unrecognized, or be mistaken for an “insect bite” or an “allergic rash.” Mini-erythema migrans are less likely to be diagnosed. Secondary erythema migrans are observed in approximately 50% of cases. Bacteriologic and pathologic analogies have been reported between tertiary neuroborreliosis and tertiary neurosyphilis (Miklossy, 2012). Syphilis, once well-known as the great imitator, gives us a good historical model for the concept of occult infection.

## Occult infections and their role in the pathophysiology of some diseases of unclear etiology

Charles Nicolle, working at the Institut Pasteur in Tunis and Nobel prize winner in 1928, showed great interest in the concept of occult infections (“les infections inapparentes”) like typhus, syphilis, and relapsing fever (*Borrelia recurrentis*) (Nicolle, 1993). Relapsing fever due to another species of *Borrelia* (*B. crocidurae*) is still a public health concern in some parts of Africa, and the recently discovered *B. miyamotoi* may also become a similar problem in Asia, Europe and America (Schwan et al., 2012; Branda and Rosenberg, 2013; Lee et al., 2014). Peptic ulcer disease is another example of the hidden link between an occult infection with another spiral-shaped bacterium, *Helicobacter pylori*, and a chronic disorder. *B. burgdorferi* may persist in tissues even after antibiotic treatments, as animal models have shown (Straubinger et al., 1997; Straubinger, 2000; Hodzic et al., 2008; Yrjänäinen et al., 2010; Embers et al., 2012). In fact dormant persister cells of bacteria from different genera can escape the bactericidal effect of antibiotics and be responsible for latent infections (Phillips et al., 1998; Hunfeld et al., 2005; Lewis, 2007; Lee et al., 2014). Clinicians have no diagnostic tests to check for the persistence of live borreliae. *B. burgdorferi*, having a complex genetic structure, is a highly adaptable organism capable of evading immune response through different processes. It can survive extracellularly and intracellularly (Brorson and Brorson, 1998; Murgia and Cinco, 2004). The complexity of Lyme disease requires high quality diagnostic methods, yet serology is the only diagnostic tool widely used.

## Serology, the current main diagnostic method

Physicians should be made aware that, in the presence of primary erythema migrans, serology will often be negative therefore diagnosis should be clinical (Wormser, 2006). However, many practitioners are still under the misconception that a positive serology is required for early stage diagnosis. For later stages of the disease serology remains the main diagnostic tool. The Infectious Diseases Society of America (IDSA) and the European Concerted Action on Lyme Borreliosis (EUCALB) are recommending a two-tier testing approach, the first step being an ELISA using whole sonicate of the *in vitro* cultured tick-derived strain B31 of *Borrelia burgdorferi* (EUCALB, 1997; Wormser et al., 2006). If positive, confirmation by immunoblot testing IgG and IgM is required. According to these guidelines, immunoblot is not to be performed if the ELISA is negative. However, in 2011, the CDC modified their case definition and included single-tier IgG immunoblot seropositivity as a diagnostic criterion for Lyme disease (CDC, 2013). But most practitioners still use the two-tier system despite the poor sensitivity of ELISA tests, ranging from 34 to 70.5 (Marangoni et al., 2005; Aguero-Rosenfeld, 2008; Ang et al., 2011; Wojciechowska-Koszko et al., 2011). Calibration of the tests is a crucial issue.

## Calibration of serology

When Lyme serology was developed, no reliable method was available to be used as a gold standard for comparison. As most of the signs and symptoms are non-specific, no reliable clinical diagnostic score could be established. The low yield of culture and the difficulty involved in using the technique routinely were another major obstacle. A pragmatic cut-off level for the serologic tests had to be determined arbitrarily on blood donors (EUCALB, 1997; Assous, 2007). In the late seventies, when Lyme disease was first discovered, it was understandably thought to be a rare and regional phenomenon. Therefore, a low prevalence was set as experts were afraid the serologies would produce too many false positive diagnoses (EUCALB, 1997; Assous, 2007). Patients and control populations are ill-defined with a high variability in predictive positive and negative values from one test to another. Culture of *B. burgdorferi* or detection of its genome by polymerase chain reaction (PCR) may occasionally confirm the clinical diagnosis in seronegative patients, however none of these methods are sensitive enough to be considered reliable diagnostic methods, especially in routine practice (Schutzer et al., 1990; Nields and Kveton, 1991; Chmielewska-Badora et al., 2006; Brunner, 2006; Assous, 2007; Holl-Wieden et al., 2007; Aguero-Rosenfeld, 2008; Dietrich et al., 2008; Wallet et al., 2008). As a result, many patients suffering signs and symptoms compatible with Lyme disease, but whose test is negative, are falling by the wayside.

## Clinical and epidemiological consequences of negative serology

Modern medical practice expects to rely on evidence. Most physicians would not consider diagnosing Lyme disease without serological proof. Yet the failure to diagnose seronegative neuroborreliosis, especially the acute or severe forms, can have dire consequences including chronic neurologic sequelae or even death. A review of the literature shows that a diagnosis of Lyme neuroborreliosis is often difficult to prove (Blanc et al., 2007; Bennet et al., 2008; Tveitnes et al., 2009; Makhani et al., 2011). The sensitivity of intrathecal antibody index (measuring specific antibodies within the cerebro-spinal fluid) ranges from 55 to 80%. In a Swedish study, antibodies were present in serum of only 23% of children with neuroborreliosis (Bennet et al., 2008). Cognitive tests or SPECT brain imaging may help to provide objective evidence (Tager et al., 2001; Roche-Lanquetot et al., 2008; Fallon et al., 2009; Donta et al., 2012). Pragmatic diagnostic criteria including response to empiric antibiotic treatment are used to diagnose neuroborreliosis (Blanc et al., 2007). Should this strategy be recommended in other clinical presentations as well? In fact some clinicians will not hesitate to classify as Lyme disease cases, seronegative patients with a highly compatible clinical picture, provided other diagnoses have been ruled out. In a major clinical trial on Lyme disease, 40% of the enrolled patients were seronegative. These patients had a history of erythema migrans, neurologic or cardiac symptoms, radiculoneuropathy or arthritis (Klempner et al., 2001). Clinicians, often unaware of the difficulties involved in diagnosing Lyme disease, will fall back on “weak” alternative diagnoses (“viral,” “idiopathic,” “auto-immune,” “degenerative,” “inflammatory,” or “psychosomatic”) (Kennedy, 2013). New techniques are needed to accurately assess these patients. This current over-reliance on inaccurate testing procedures not only flaws the diagnosis of individual patients but it also has epidemiological consequences especially as new species and variants continue to be identified on all continents (Hao et al., 2011; Rudenko et al., 2011).

## Possible causes of seronegativity

Several factors leading to seronegativity have been identified in confirmed cases of Lyme disease: (i) the arbitrary cut-off level of tests, (ii) the sequestration of antibodies in immune complexes, (iii) the wide variety of species and subspecies of *Borrelia* that co-exist in different parts of the world, and (iv) coinfections with other pathogens which may be responsible for some or all of the symptoms or which may alter the immune response (Schutzer et al., 1990; Brunner, 2006). The complex *B. burgdorferi sensu lato* includes (Table 1): *B. burgdorferi sensu stricto* (including genetic diversity), *B. afzelii, B. garinii* (several serotypes) and additional species isolated in different parts of the world (Rudenko et al., 2009, 2011; Ogden et al., 2011). Some of these species have been isolated in symptomatic patients (Varela et al., 2004; Lopes de Carvalho et al., 2009; Rudenko et al., 2009; Stanek and Reiter, 2011; Branda and Rosenberg, 2013; Clark et al., 2013; Lee et al., 2014; Margos et al., 2014). *B. spielmanii* may cause early skin disease (Stanek and Reiter, 2011). *B. bavariensis, B. bisettii, B. valaisiana, B. americana, B. andersonii, B. lonestari* and more recently *B. kurtenbachii* have been isolated from patients with Lyme-like diseases (Varela et al., 2004; Rudenko et al., 2009; Rizzoli et al., 2011; Stanek and Reiter, 2011; Clark et al., 2013). The pathogenic role of *B. lusitaniae*, isolated in a case of vasculitis, remains to be substantiated (Rudenko et al., 2009). Despite such diversity in strains, most of the commercially available tests still rely on the original 1982 Massachusetts B31 isolate of *B. burgdorferi*. No diagnostic tool is available for routine detection of *B. miyamotoi* (Branda and Rosenberg, 2013; Lee et al., 2014). Coinfections with other microbes add to the complexity of these illnesses (Table 1). Among patients with early Lyme disease in the USA, 2–12% were found to also have human granulocytic anaplasmosis, and 2–40% babesiosis (Wormser et al., 2006). In Brazil, a Lyme-like syndrome, due to the tick *Amblyomma*, has been described and mobile non cultivable spirochetes could be visualized in patients' blood using a dark field microscope (Mantovani et al., 2007). A new tick-borne bacterial pathogen, *Candidatus* Neoehrlichia mikurensis, was reported in Switzerland (Fehr et al., 2010). An illustration of the limits of serology is the Scottish example: the sensitivity of the immunoblot was improved by using local Scottish strains of *Borrelia* (Mavin et al., 2007, 2009).

**Table 1:** **Bacteria responsible for Lyme or Lyme-like disease and other *Borrelia* sp. belonging to the *Borrelia burgdorferi sensu lato* complex, and other tick-borne micro-organisms isolated in humans**.

**Bacteria responsible for Lyme disease belonging to the *Borrelia burgdorferi sensu lato* complex**	
*Borrelia burgdorferi sensu stricto* (including genetic diversity)	North America, Europe, North Africa
*Borrelia afzelii*	Europe, Asia
*Borrelia garinii* (several serotypes)	Europe, Asia, North Africa
*Borrelia bavariensis* (previously *B. garinii* OspA serotype 4)	
**Bacteria responsible for Lyme-like disease**	
*Borrelia lonestari*	North America
*Borrelia miyamotoi* (also cause of relapsing fever)	Europe, Asia, North America
Non-identified spirochete	Brazil
**Bacteria occasionally isolated in cases of Lyme-like disease**	
*Borrelia spielmanii*	*Borrelia bisettii*
*Borrelia andersonii*	*Borrelia valaisiana*
*Borrelia americana*	*Borrelia kurtenbachii*
Novel *Borrelia sp.* close to relapsing fever borreliae (Lee et al., 2014)	
**Other *Borrelia sp.*, belonging to the *Borrelia burgdorferi sensu lato* complex with unknown or poorly documented pathogenicity**	
*Borrelia japonica*	*Borrelia turdi*
*Borrelia sinica*	*Borrelia tanukii*
*Borrelia lusitaniae* (vasculitis?)	*Borrelia californiensis*
*Borrelia carolinensis*	*Borrelia yangtze*
***Borrelia sp.* responsible for relapsing fever**	
**Louse-borne relapsing fever**	
*Borrelia recurrentis*	
**Tick-borne relapsing fever**	
At least 15 *Borrelia sp.* including	
Borrelia crocidurae (Africa)	
*Borrelia miyamotoi* (also cause of Lyme-like disease)	
**Other human tick-borne infections**	
**PARASITES**	
*Babesia divergens*	*Babesia microti*
**BACTERIA**	
*Ehrlichia chaffeensis*	*Anaplasma phagocytophilum*
*Rickettsia sp.*	*Coxiella burnetii*
*Francisella tularensis*	*Candidatus* Neoehrlichia mikurensis
**VIRUSES**	
Several *Flaviviridae* (including Tick-borne encephalitis virus)	
*Bunyaviridae* (Crimean-Congo hemorrhagic fever)	

## Conclusion and perspectives

The numerous complexities of Lyme disease make it an extremely difficult illness to fully comprehend. It remains a diagnostic challenge even for the best informed of clinicians. The lack of a gold standard for diagnosis renders the management of patients difficult and seriously hinders our ability to produce accurate statistics, especially as very similar syndromes could be due to other species of *Borrelia*. In some patients suffering from syndromes of unclear origin, following tick bite, other microbial agents could also be playing a role. Lyme disease has now entered the political debate as shown by the amendment (Section 54.1-2963.2) voted in 2013 by the State of Virginia, USA, that compels physicians to inform their patients that the “current laboratory testing for Lyme disease can be problematic.” The fact that politicians are being called upon to rule on these matters should prompt scientists to regain control of the situation. Politicians should instead become aware of the necessity to fund research and facilitate the setting up of independent international working groups. Reliable testing is essential to investigate the many syndromes of unclear origin that may mimic many other medical disorders. Proper fundamental and clinical research is urgently needed as it would be the most cost effective way of ensuring that patients are accurately diagnosed and that the best therapeutic strategies are decided upon (Stricker and Johnson, 2014). Development of new diagnostic methods is badly needed. New PCR methods and new genomic techniques, such as high throughput sequencing, could prove promising in identifying the complex mix of microbial agents that are probably involved (Vayssier-Taussat et al., 2013; Lee et al., 2014). Next generation sequencing allowed the identification of various bacteria from *Ixodes ricinus* ticks in France: *Anaplasma phagocytophilum, Bartonella henselae, B. grahamii, Borrelia afzelii, B. garinii, B. burgdorferi, B. miyamotoi, Candidatus* Neoerlichia mikurensis, *Ehrlichia canis, Rickettsia canadensis, R. felis*, and *R. helvetica* (Vayssier-Taussat et al., 2013). These new techniques should be applied to human samples. Other variables, such as genetic, environmental, or auto-immune factors should also be studied. The name “Lyme disease” is too restrictive as it focuses and fuels the controversy. A new term should be agreed upon for these syndromes with possible infectious involvement, often following tick bites. Closer collaboration between epidemiologists, microbiologists, immunologists, geneticians, environmental scientists, veterinarians, entomologists, and clinicians is needed to identify the main agents that could be causing these occult infections and to determine strain pathogenicity. A new multidirectional approach is crucial in order to widen the field of research and to move forward.

### Conflict of interest statement

The author declares that the research was conducted in the absence of any commercial or financial relationships that could be construed as a potential conflict of interest.
